# Modulation of doxorubicin-induced expression of the multidrug resistance gene in breast cancer cells by diltiazem and protection against cardiotoxicity in experimental animals

**DOI:** 10.1186/s12935-019-0912-0

**Published:** 2019-07-24

**Authors:** Hamdan S. Al-malky, Abdel-Moneim M. Osman, Zoheir A. Damanhouri, Huda M. Alkreathy, Jumana Y. Al Aama, Wafaa S. Ramadan, Ali A. Al Qahtani, Hadiah B. Al Mahdi

**Affiliations:** 1 Pharmacology Department, Faculty of Medicine, KAU, Jeddah, Saudi Arabia; 2 Pharmacology Unit, National Cancer Institute, Cairo University, Cairo, Egypt; 3 Department of Genetic Medicine, Faculty of Medicine, KAU, Jeddah, Saudi Arabia; 4 Princess Aljawhara Center of Excellence in Research of Hereditary Disorders, KAU, Jeddah, Saudi Arabia; 5 Anatomy Department, Faculty of Medicine, KAU, Jeddah, Saudi Arabia; 6 Anatomy Department, Faculty of Medicine, Ain Shams University, Cairo, Egypt

**Keywords:** Doxorubicin, Diltiazem, MCF-7 cells, MDR, Cardiotoxicity

## Abstract

**Background:**

Doxorubicin (DOX) is one of the most important anticancer agents used in treating breast cancer. However, chronic cardiotoxicity and multidrug resistance limit the chemotherapeutic use of DOX.

**Methods:**

This study aimed to evaluate the capability of calcium channel blocker diltiazem (DIL) to reverse DOX resistance in breast cancer MCF-7 cells and to confer protection against DOX-induced cardiotoxicity in Wistar rats. For this purpose, we explored the effects of DOX on cell cycle phase distribution and expression of ABCB1, FOXO3a, and p53 genes in the presence and absence of DIL (20 μg/ml) and studied the ability of DIL to prevent DOX-induced cardiotoxicity after a single injection of DOX (15 mg/kg) in male Wister rats.

**Results:**

We found that compared with DOX alone treatment, DIL + DOX treatment down regulated the ABCB1 gene expression by > fourfold but up regulated the FOXO3a and p53 genes expression by 1.5 fold. DIL treatment conferred protection against DOX-induced cardiotoxicity, as indicated by a decrease in the levels of the cardiac enzyme creatine kinase MB and malondialdehyde and an increase in the total antioxidant capacity and glutathione peroxidase levels. These biochemical results were further confirmed by the histopathological investigation of cardiac cells, which showed normal cardiac cells with central vesicular nuclei and prevention of DOX-induced disruption of normal cardiac architecture in the DIL to DOX group.

**Conclusions:**

Taken together, our results indicate that DIL treatment can reverse the resistance of breast cancer cells to the therapeutic effects of DOX and can protect against DOX-induced cardiotoxicity in rats.

## Background

Multidrug resistance (MDR) continues to be a major clinical obstacle to the effects of cancer chemotherapy. MDR cancer cells are resistant to the naturally occurring cancer drugs, such as epipodophyllotoxins, vinca alkaloids, and anthracyclines, but are not cross-resistant to antimetabolites, alkylating agents, and cisplatin [1]. Doxorubicin (DOX) is a cytotoxic anthracycline antibiotic used for treating various types of cancers, such as Hodgkin and non-Hodgkin’s lymphoma, multiple myeloma, sarcoma, pediatric cancers, and breast, lung, ovarian, and thyroid cancers [2]. Several studies have confirmed the superiority of the regimens containing anthracyclines over those lacking them [3, 4]. As with other anticancer agents, the clinical use of DOX is hindered by tumor resistance and toxicity to healthy tissues [5]. Resistance to this drug is common, thus representing the main obstacle to the effective treatment of the disease [6, 7]. To counter MDR to the effects of breast cancer therapeutics, a wide range of compounds capable of inhibiting the MDR gene have been studied. Such inhibitors include, but are not limited to, the anti-HIV protease inhibitors ritonavir and nelfinavir, fumitremorgin C, and biochanin A. The major side effect of DOX is cardiotoxicity; therefore, strategies that minimize this side effect are urgently needed. Diltiazem (DIL) is a calcium channel blocker (CCB) that is widely used in the treatment of various indications. Cornwell et al. [8] reported that DIL, along with other CCBs, reverses MDR by binding to membrane vesicles and proteins associated with MDR development in tissue culture cells. Therefore, our study focused on investigating the molecular mechanisms underlying DIL-induced enhancement in the cytotoxic activity of DOX by measuring the expression of genes responsible for drug resistance, apoptosis induction, and cell cycle disturbance. Moreover, we evaluated the protective effect of DIL against DOX-induced cardiotoxicity in Wistar rats by evaluating total antioxidant capacity (TAC) and the levels of creatine kinase-MB (CK-MB), glutathione peroxidase (GPx), and malondialdehyde (MDA) and by performing histopathological investigation of cardiac tissue.

## Materials and methods

### Drugs and chemicals

DOX, DIL and Trypan blue powder were purchased from Sigma Aldrich Co. (Saint Louis, Missouri, USA) while Verapamil was purchased from Abbott. Fetal bovine serum (FBS), Dulbecco’s Modified Eagle Medium (DMEM), Trypsin–EDTA (0.05%) and phosphate buffer saline (PBS, pH 7.4) were purchased from Thermo Fisher Scientific Inc (USA). Acridine orange (AO) (Molecular Probes, Eugene, OR, cat. no. A1301) and Giemsa stain (GS-10), rhodamine 123 (R8004 SIGMA) have been also supplied. The cell cycle determination kit was purchased from Cayman Chemical Company (USA). Animal kits include Rat CK-MB (Catalog No: E-EL-R1327 (USA)), MDA (Catalog No: E-EL-0060 (USA)), TAC (Catalogue No. 201-11-1187 (USA)) and GPx (Catalog No: CAT. No. GP 2524 (Biodiagnostic, Dokki, Egypt).

### Cells and cell cultures

For use in this study, human breast cancer cell line MCF-7 was obtained from the National Cancer Institute, Cairo University, Egypt. The adherent cells were grown as a monolayer in DMEM supplemented with penicillin (100 IU/ml), streptomycin (100 µg/ml), and 10% FBS. Cells were cultured at 37 °C in a humidified 5% CO_2_ atmosphere and were passaged every 4–5 days.

### Cell cycle analysis

Cells were seeded in six-well plates at a density of 10^5^–10^6^ cells/well in DMEM supplemented medium and incubated in a CO_2_ incubator at 37 °C for at least 24 h. DOX at 0.25 or 1 μg/ml concentration and/or DIL at 20 μg/ml concentration were then added, followed by incubation for additional 48 h. Subsequently, the cell medium was removed, and the cells were washed with PBS and harvested with trypsin/ethylenediaminetetraacetic acid (EDTA). Following trypsinization, cells were washed twice with an assay buffer. The cell pellet was re-suspended in the assay buffer to a density of 1 × 10^6^ cells/ml. Subsequently, 1 ml of a fixative agent was added to each sample to fix and permeabilize the cells for at least 2 h prior to propidium iodide (PI) staining. Fixed cells were centrifuged at 500×*g* for 5 min, and the fixative agent was decanted thoroughly. The cell pellet was then suspended in the PI staining solution and incubated for 30 min at RT in the dark. Cell cycle analysis was performed using BD FACS Aria III flow cytometer (BD, San Jose, CA, USA) as per the method described by Sulic et al. [9].

### RNA isolation, cDNA synthesis, and reverse transcription quantitative PCR (RT-qPCR)

TaqMan gene expression assay was performed for ABCB1, FOXO3a, and p53 genes using a standard real-time PCR (Applied Biosystems, Foster City, CA, USA). The amplification was performed using TaqMan probe, RNA isolation, cDNA synthesis, and RT-qPCR.

Cells were seeded in six-well plates at a density of 10^5^–10^6^ cells/well in DMEM supplemented medium and were cultured in a CO_2_ incubator at 37 °C for at least 24 h. Cells were then incubated with DOX at 0.25 or 1 µg/ml concentration and/or DIL at 20 µg/ml concentration for additional 48 h. Subsequently, the cell medium was removed, and the cells were washed with PBS. Total RNA was isolated from cultured cells using the QIAamp RNA mini kit (Catalog no. 52304; Qiagen, Germany) according to the manufacturer’s instructions. RNA samples (1 μg) were reverse transcribed to cDNA using the High-Capacity RNA-to-cDNA Kit (Catalog no. 4387406; Applied Biosystems, Foster City, CA, USA) according to the manufacturer’s instructions. RT-qPCR was performed on an Applied Biosystems 7500 Fast Real-Time PCR System using TaqMan^®^ Gene Expression assay. TaqMan probes for ABCB1 gene (Assay ID: Hs00184500_m1), FOXO3a gene (Assay ID: Hs00818121_m1), TP53 gene (Assay ID: Hs01034249_m1), and the housekeeping geneRNA18S5 (Assay ID: Hs03928990_m) were obtained from Applied Biosystems. Data were analyzed, and amplification plots were generated using the 7500 Fast Real-Time PCR software. The comparative CT method (2^−ΔΔCT^) was used for relative quantification of the target gene as follows: ΔΔCT = (CT of the target gene −CT of RNA18S5) for χ − (CT of the target gene −CT of RNA18S5) for y, where χ = treated sample and y = control sample. After validation of the method, results for each sample were expressed in N-fold changes in χ target gene copies normalized to RNA18S5 relative to the copy number of the target gene in control according to the following equation: amount of target = 2^−ΔΔCT^.

### Gene expression data analysis

Data were analyzed, and amplification plots were generated using the 7500 Fast Real-Time PCR software. The comparative CT method (2^−ΔΔCT^) was used for relative quantification of the target gene as follows: ΔΔCT = (CT of the target gene −CT of RNA18S5) for χ − (CT of the target gene –CT of RNA18S5) for y, where χ = treated sample and y = control sample. After validation of the method, the results for each sample were expressed in N-fold changes in χ target gene copies normalized to RNA18S5 relative to the copy number of the target gene in control according to the following equation: amount of target = 2^−ΔΔCt^. Statistical analysis was performed using Statistical Package for the Social Sciences (SPSS) version 21. p values of ≤ 0.05 were considered statistically significant.

### Evaluation of DOX-induced cardiotoxicity in the presence of DIL

Fifty-six male Wistar rats were divided into four equal groups of 14 animals. Group I received normal saline i.p. (0.5 ml/200 gm) and was reserved as the control group. Group II received DIL (4 mg/kg body weight, i.p.). Group III received DOX (15 mg/kg body weight, i.p.). Group IV received both DIL (4 mg/kg body weight, i.p.) and DOX (15 mg/kg body weight, i.p.) simultaneously. At the end of the experiment period (48 or 72 h), rats were anesthetized to collect blood samples from the ophthalmic artery in the orbital rim; the blood samples were rapidly centrifuged for serum separation and were stored at − 80 °C until further use for evaluating cardiac CK-MB level, MDA level, TAC, and GPx level using commercial kits.

### Histopathological examination

The rats were sacrificed by decapitation, and their hearts were extracted immediately by opening their chest. Heart samples were immediately washed with saline, and part of the left ventricle was fixed in 10% phosphate-buffered formalin for 48 h and prepared for light microscopy.

### Statistical analysis

Statistical analysis was performed using SPSS version 21. One-way analysis of variance followed by least significant difference for post hoc analysis and was performed for multiple comparisons. Statistical significance was considered at P values of ≤ 0.05.

## Results

### Effect of DOX and/or DIL treatment on cell cycle phase distribution of MCF-7 cells

**Fig. 1:**
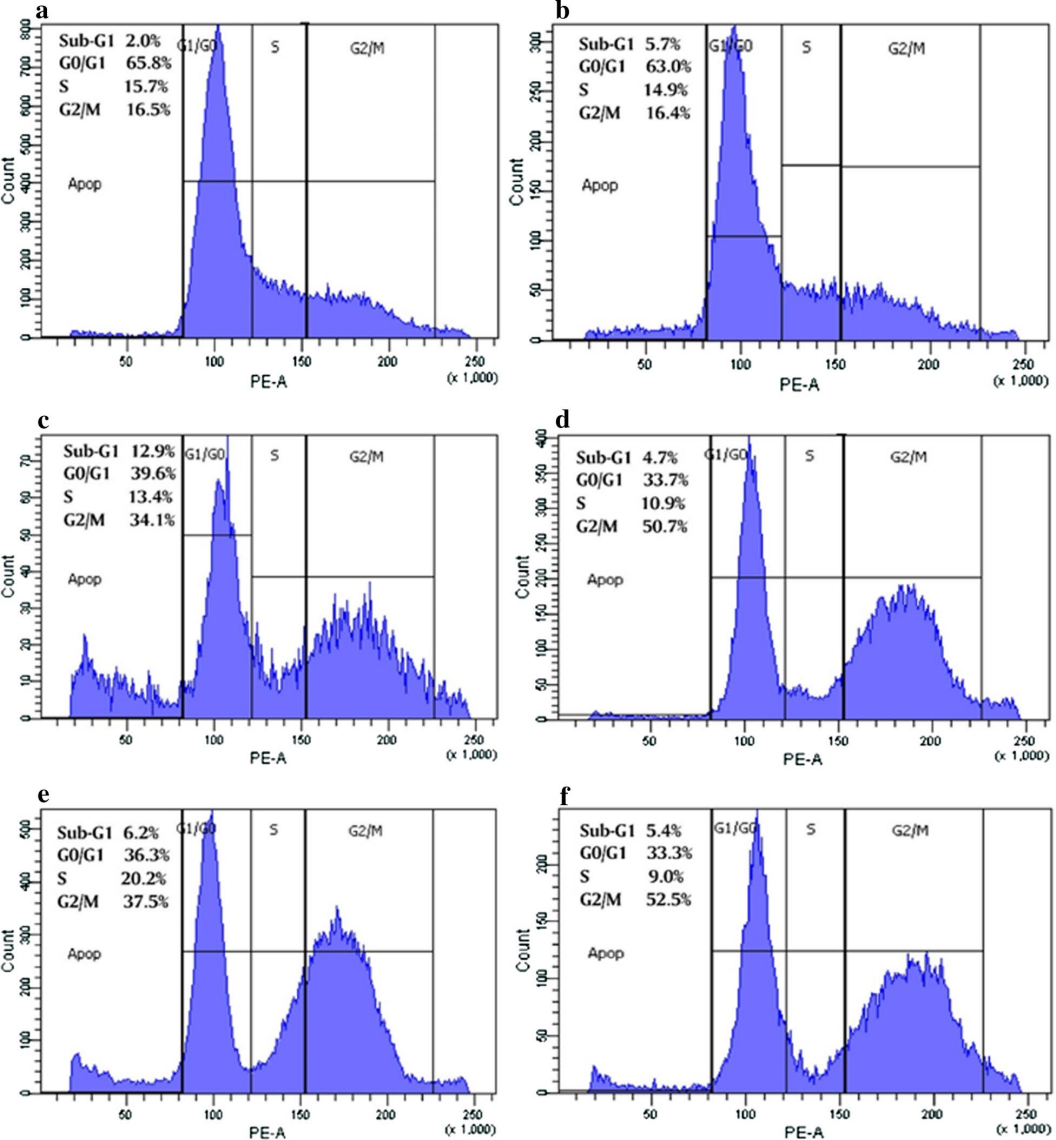
Effect of DOX and/or DIL treatment on cell cycle phase distribution of MCF-7 cells. Cell cycle distribution was analyzed by exposing the cells to drugs for 48 h, then staining with PI. **a** Control, **b** cells treated with 20 µg/ml DIL, **c** cells treated with 0.25 µg/ml DOX, **d** cells treated with 0.25 µg/ml DOX and 20 µg/ml DIL, **e** cells treated with 1 µg/ml DOX, **f** cells treated with 1 µg/ml DOX and 20 µg/ml DIL. The experiment was repeated twice each one in duplicate

Figure 1 shows the effect of 0.25 and 1 μg/ml DOX and/or 20 μg/ml DIL on cell cycle phase distribution assessed using flow cytometry after staining the cells with PI. We found that 0.25 and 1 μg/ml DOX treatment resulted in 34.1% and 37.5% accumulation of cell population in the G2/M phase, respectively, compared with the control. This cell accumulation in the G2/M phase significantly increased after the addition of 20 μg/ml DIL to DOX treatment (both concentrations).

### Evaluation of ABCB1 gene expression in MCF-7 cells by RT-qPCR

**Fig. 2:**
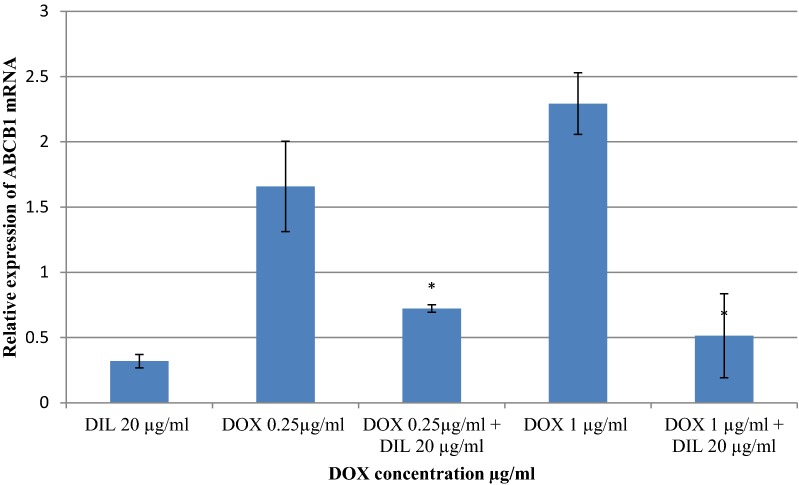
Evaluation of ABCB1 gene expression in MCF-7 cells by RT-qPCR. The delta–delta CT method was used to determine the fold change for ABCB1 gene relative expression in MCF-7 cells that were treated with DOX and/or DIL for 48 h. The values represent the mean ± SD (n = 2). *Significantly different from corresponding DOX at p-value < 0.05

Figure 2 shows the relative expression of ABCB1 mRNA by RT-qPCR. ABCB1 was over expressed in MCF-7 cells after treatment with 0.25 and 1 μg/ml DOX. This over expression of ABCB1 mRNA decreased by 2.3 and 4.5 fold, respectively, after the addition of 20 μg/ml DIL, indicating that the addition of DIL reversed ABCB1/P-gp-mediated MDR.

### Evaluation of FOXO3 gene expression in MCF-7 cells

**Fig. 3:**
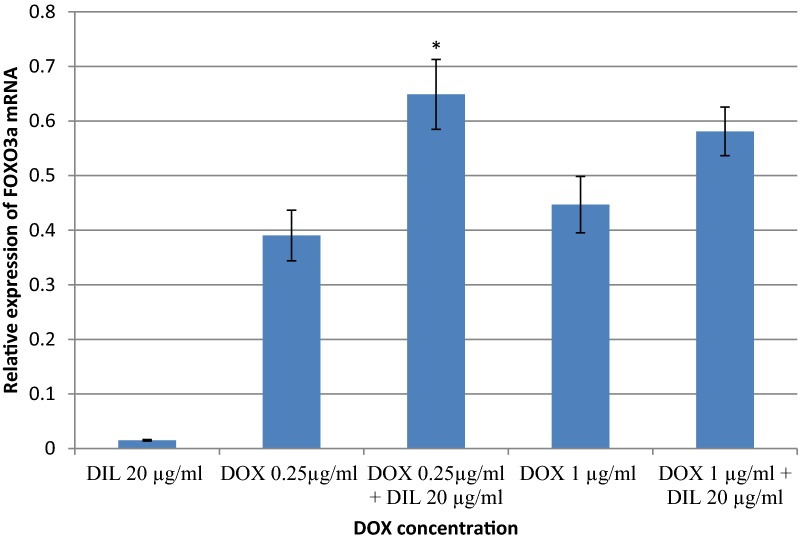
Evaluation of FOXO3a gene relative expression in MCF-7 cells by RT-qPCR. The delta–delta CT method was used to determine the fold change for FOXO3a gene expression in MCF-7 cells that were treated with DOX and/or DIL for 48 h. The values represent the mean ± SD (n = 2). *Significantly different from corresponding DOX at p-value < 0.05

Figure 3 shows the relative expression of FOXO3a mRNA by RT-qPCR. The relative expression of FOXO3a in MCF-7 cells was 0.39 and 0.45 after treatment with 0.25 and 1 μg/ml DOX, respectively. This expression increased by 1.7 and 1.3 fold to 0.65 and 0.58, respectively, after the addition of 20 μg/ml DIL.

### Evaluation of p53 gene expression in MCF-7 cells

Table 1 shows the relative expression of p53 by RT-qPCR. P53 was over expressed in MCF-7 cells after treatment with 0.25 and 1 μg/ml DOX. This expression increased by 1.4 and 1.5 fold, respectively, after the addition of 20 μg/ml DIL.

**Table 1:** Evaluation of p53 gene expression in MCF-7 cells

Treatment	Relative expression of p53
DIL 20 µg/ml	1.06 ± 0.65
DOX 0.25 µg/ml	1.20 ± 0.64
DOX 0.25 µg/ml + DIL 20 µg/ml	1.71 ± 1.4
DOX 1 µg/ml	1.43 ± 1.5
DOX 1 µg/ml + DIL 20 µg/ml	2.21 ± 1.1^a^

Relative p53 mRNA gene expression level by RT-qPCR in MCF-7 cells that were treated with DOX/and or DIL for 48 h. The values represent the mean ± SD

^a^Significantly different from corresponding DOX at p-value < 0.05 (the delta–delta CT method)

### Evaluation of CK-MB levels

Table 2 shows the effect of DOX and/or DIL treatment on serum CK-MB levels in rats. After 48 and 72 h of DOX treatment, the CK-MB levels were significantly increased in rats by 6.7 and 5.8 fold, respectively, compared with the control. These CK-MB levels decreased significantly by 1.8 and twofold, respectively, after the addition of DIL.

**Table 2:** Effect of DOX and/or DIL on serum CK-MB level

Treatment	CK-MB (pg/ml)	
	(48 h)	(72 h)
Control	103 ± 14	112 ± 11
DIL (4 mg/kg i.p.)	100 ± 10	104 ± 21
DOX (15 mg/kg, i.p.)	690 ± 38^a^	659 ± 48^a^
DOX + DIL (administrated simultaneously)	367 ± 87^b^	348 ± 19^b^

Data are expressed as mean ± SD of experiment in male Wistar rats after 48 h and 72 h (n = 2)

^a^Significantly different from control at p-value < 0.05

^b^Significantly different from corresponding DOX at p-value < 0.05

### Evaluation of MDA levels

Table 3 shows the effect of DOX and/or DIL treatment on the MDA levels in rats. After 48 and 72 h of DOX treatment, the MDA levels were significantly increased in rats by 2.5 and threefold, respectively, compared with the control. The addition of DIL to DOX treatment restored the MDA levels to nearly the normal level.

**Table 3:** Effect of DOX and/or DIL on MDA level

Treatment	MDA (ng/ml)	
	(48 h)	(72 h)
Control	53 ± 17	52 ± 4
DIL (4 mg/kg i.p.)	74 ± 16	73 ± 6
DOX (15 mg/kg, i.p.)	138 ± 21^a^	152 ± 31^a^
DOX + DIL (administrated simultaneously)	76 ± 42^b^	99 ± 2^b^

Data are expressed as mean ± SD of experiment in male Wistar rats after 48 h and 72 h (n = 2)

^a^Significantly different from control at p-value < 0.05

^b^Significantly different from corresponding DOX at p-value < 0.05

### Evaluation of total antioxidant capacity

Table 4 shows the effect of DOX and/or DIL treatment on TAC levels in rats. After 48 and 72 h of DOX treatment, the TAC levels were significantly decreased in rats by 52% and 43%, respectively, compared with the control. The addition of DIL to DOX treatment restored the TAC levels to nearly the normal level.

**Table 4:** Effect of DOX and/or DIL on serum TAC

Treatment	Total antioxidant capacity (U/ml)	
	(48 h)	(72 h)
Control	27 ± 12	28 ± 3
DIL (4 mg/kg i.p.)	28 ± 3	30 ± 5
DOX (15 mg/kg, i.p.)	13 ± 7^a^	16 ± 2^a^
DOX + DIL (administrated simultaneously)	31 ± 5^b^	30 ± 1^b^

Data are expressed as mean ± SD of 2 experiments in male Wistar rats after 48 h and 72 h

^a^Significantly different from control at p-value < 0.05

^b^Significantly different from corresponding DOX at p-value < 0.05

### Evaluation of serum GPx levels

Table 5 shows the effect of DOX (15 mg/kg, i.p.) and/or DIL (4 mg/kg, i.p.) on serum GPx levels in rats. After 48 and 72 h of DOX treatment, the GPx levels significantly decreased in rats by 1.8 and twofold, respectively, compared with the control. The addition of DIL to DOX treatment restored the GPx levels to the normal level.

**Table 5:** Effect of DOX and/or DIL on serum GPx level

Treatment	Glutathione peroxidase (U/ml)	
	(48 h)	(72 h)
Control	9.6 ± 0.48	9.7 ± 0.72
DIL (4 mg/kg i.p.)	9.8 ± 0.72	10.1 ± 0.46
DOX (15 mg/kg, i.p.)	5.4 ± 0.33^a^	4.8 ± 0.91^a^
DOX + DIL (administrated simultaneously)	9.4 ± 0.86^b^	9.5 ± 0.42^b^

Data are expressed as mean ± SD of 2 experiments in male Wistar rats after 48 h and 72 h

^a^Significantly different from corresponding control at p-value < 0.05

^b^Significantly different from corresponding DOX at p-value < 0.05

### Histopathological investigation of heart tissue after DOX and/or DIL treatment

**Fig. 4:**
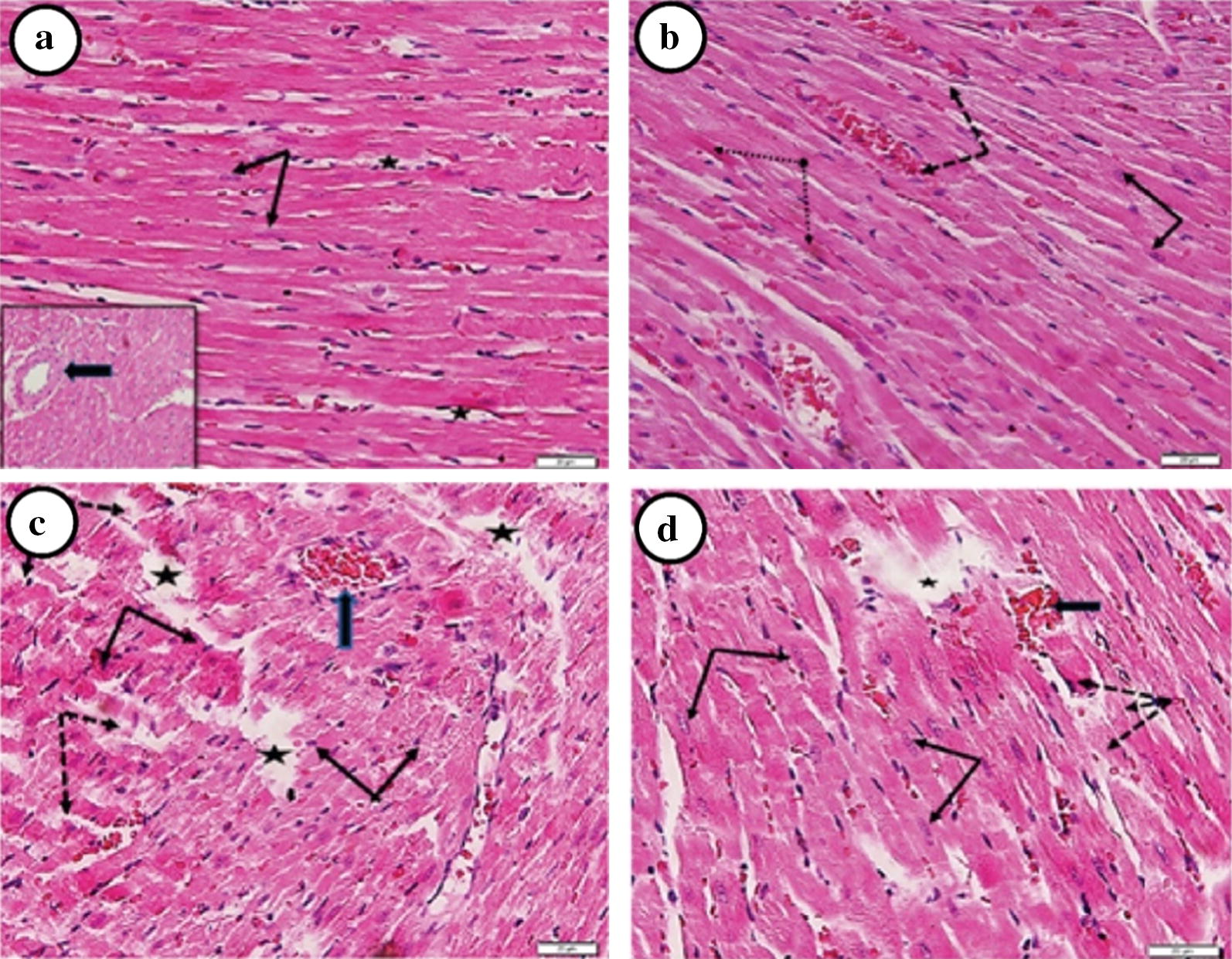
Photomicrograph of a section of a rat heart of **a** control group. Showing a regular pattern of branching cardiac myocytes (arrows). Capillaries are noted between the myocytes (  ). **b** DIL (4 mg/kg) given 2 h before DOX 15 (mg/kg). Showing well organized architecture of branching cardiac muscle fibers with oval vesicular nuclei (arrows). Dilated, congested capillaries between muscle fibers are noted (dashed arrows). **c** DOX 15 mg/kg. Showing marked disruption of normal cardiac architecture. Multiple areas of fragmented cardiac muscle fibers (dashed arrows), zones of complete loss (  ) and hemorrhages (thick arrows) are noted. **d** DOX 15 (mg/kg) and DIL (4 mg/kg). Showing normal branching cardiac muscle fibers with central vesicular nuclei (arrows). Localized areas of shortened cardiomyocytes having deep eosinophilic cytoplasm and condensed peripheral nuclei (dashed arrows) or focal areas of loss (  ) are still revealed (H&E ×400)

DIL treatment alone (Fig. 4b) showed well-formed architecture of cardiac muscle fibers picture like control (Fig. 4a). Light photomicrographs (Fig. 4c) showed the effect of DOX (15 mg/kg) treatment on the myocardium tissue of rats. DOX treatment showed a marked disruption of normal cardiac architecture, congestion of blood vessels and capillaries, condensed pyknotic peripheral nuclei and multiple areas of fragmented cardiac muscle fibers. The histopathological changes induced by DOX were less when combined with DIL therapy. The Combination of DOX (15 mg/kg) and DIL (4 mg/kg) (Fig. 4d) showed normal branching cardiac muscle fibers with central vesicular nuclei with normal cardiomyocytes.

## Discussion

DOX is an anthracycline antibiotic with a wide spectrum of antitumor activity. It is commonly used in treating different cancers. Unfortunately, in addition to chemo-resistance that may affect DOX against tumors. In the other part using DOX may cause chronic cardiotoxicity and cardiomyopathy development which is the main factor that limits the chemotherapeutic uses of DOX [10]. Resistance to chemotherapeutic drugs is one of the important reasons for cancer recurrence, progression, and metastasis. Our study focused on investigating whether DIL can overcome the resistance to DOX therapy and enhance the cytotoxic effects of DOX against the growth of MCF-7 human breast cancer cell line. In this case, we studied the possible modulatory mechanisms changes in cell cycle phase distribution, the molecular mechanism of inhibition of ABCB1 gene expression responsible for drug transport as well as expression of the tumor suppressor genes FOXO3a and p53. In the second part of this study, we also investigated the possible protection conferred by DIL against DOX-induced cardiotoxicity in Wistar rats through many indicators that targeted the oxidative stress.

It is well known that DNA damage caused by different cytotoxic agents, induced cell cycle arrest at G1, S, G2, thereby preventing replication of damaged DNA or aberrant mitosis which if not repaired, may result in either tumorigenesis or apoptosis [11]. It has been justified based on the fact that anthracyclines are mostly active on proliferating cells in G2/M phase due to the maximal expression of their target enzyme TOPO II at these phases [12, 13].

In our study, the results showed that DIL treatment induced accumulation of G2/M phase by approximately threefold when added to both DOX concentrations compared to control and by 1.4 fold compared to the corresponding DOX-treated cells administered alone. In general, CCBs have been demonstrated to induce apoptosis, decrease cellular proliferation indices, and decrease metastatic potential and invasion in many cancer cell lines [14, 15]. In the majority of these studies, the authors were unable to demonstrate that this effect resulted due to inhibiting the calcium-dependent secondary messenger systems within these cells.

The other problem that affects the cytotoxicity effect of DOX is MDR development and subsequent relapse on chemotherapy, but unfortunately, the underlying molecular processes remain poorly understood [16]. In general, ATP-binding cassette (ABC) transporters play important roles in MDR in breast cancer; an increase in ABC expression results in a reduced response to a range of chemotherapeutic drugs and the eventual reduction in survival among breast cancer patients [17].

We showed that ABCB1 mRNA was over expressed in MCF-7 cells after DOX treatment, possibly leading to the drug resistance (Fig. 2). On the other hand, DIL treatment was found to reverse ABCB1/P-gp mediated MDR, as shown by > fourfold decrease in ABCB1 mRNA expression after the addition of DIL. In fact, this down-regulation re-sensitized MCF-7 cells to DOX treatment and confirmed that ABCB1 protein expression was knocked down by DIL treatment, leading to possible increased DOX uptake and cytotoxicity. Similar to our study, Komoto et al. assessed the effects of 12 Ca^2+^ antagonists, including DIL, on MDR1 mRNA expression in human cervical carcinoma (HeLa and Hvr100-6 cells) previously treated with chemotherapeutic agents to find a novel strategy to reverse MDR1-mediated MDR [18]. Consistent with our findings, they found a significant reduction in MDR1 mRNA expression after DIL treatment but variable results for other Ca^2+^ antagonists, confirming that the down-regulation of MDR1 mRNA expression contributed to the reduction in resistance via increases in the intracellular concentrations of anticancer drugs. Moreover, Didziapetris et al. and Genovese et al. reported that DIL is an ABCB1 substrate that may inhibit ABCB1 expression [19, 20].

In addition, our result showed a 1.5 fold increase in FOXO3a expression, a tumor suppressor gene, after addition of DIL to DOX treatment (Fig. 3). This expression may be considered responsible for the increase in the cytotoxic activity of DOX. It is consistent with that of Farhan et al. who proposed that FOXO3a activation promotes cell cycle arrest and cancer cell apoptosis, which is beneficial for treating cancer [21]. It has been suggested that drugs that activate FOXO3a can be used in combination with other therapeutic agents to sensitize tumor cells [21, 22]. In the same manner, our present study also showed that the expression of the tumor suppressor gene p53 was increased after DOX treatment; this expression further increased by 1.5 fold after the addition of DIL (Table 1). Many studies have shown that the p53 signaling pathway is essential for DOX-induced cytotoxicity in cancer cells as DOX exerts its anticancer function by inducing cell cycle arrest and apoptosis in cancer cells [23, 24]. Dziegielewska et al. reported that epithelial tumor cells express T-type Ca^2+^ channels, which are thought to promote cell proliferation and has more response to T-type Ca^2+^ channel inhibition. Selective T-type Ca^2+^ channel antagonists caused growth inhibition and apoptosis more effectively in the colon cancer cell (HCT116 cells) that expressed wild-type p53 [25].

The second part of this study aimed to study the protective effect of DIL against DOX cardiotoxicity in Wistar rats. As mentioned that the cardiotoxicity either acute or chronic is the major limiting complication of DOX treatment [5]. The mechanisms underlying the development of DOX-induced cardiotoxicity has poorly understood, although activation of several cellular pathways has been proposed, including the local release of vasoactive substances, mitochondrial dysfunction, lipid peroxidation and glutathione peroxidase depletion [26, 27]. In addition, much recent attention has been focused on the potential involvement of myocardial ROS, which is increased by DOX and modulate several of the key remodeling processes [28].

In the present study, the DOX-induced acute cardiotoxicity was indicated by the changes in the serum levels of total CK-MB (Table 2). It is well known that this enzyme is released from the heart muscle cells when they are injured and that its levels in the blood after myocardial injury reflect the extent of damage to the cardiac musculature. In addition, the MDA levels and TAC were variable and different from the normal levels. These results are in a good agreement with those of previous studies on DOX-induced cardiotoxicity that the oxidative stress was evident by the reduction in serum TAC (Table 4) and GPx levels (Table 5) and the increase in lipid peroxidation indicated by increased serum MDA levels (Table 3).

Due to its cardiotoxicity, various strategies have been employed to prevent this series of an adverse effect such as the use of combination treatment, cardioprotective and synthesis of modified anthracyclines [29]. In this study, the addition of DIL to DOX treatment resulted in > twofold increase in the GPx levels and TAC and a twofold decrease in the MDA levels. These results are in good agreement with others who reported cardiac toxicity after DOX [30, 31]. They have shown a decreased serum level of TCA and increased the level of MDA after DOX administration in rats. Our data are also in a consistent with Feridooni et al. who found potential prevention or attenuation of anti-cancer drug-induced cardiotoxicity (Irinotecan and DOX) by using anti-ischemic drugs (losartan and DIL) plus dexrazoxane in (H9c2) rat cardiomyoblast cell line. They found that losartan and DIL were as effective as dexrazoxane in protecting the cardiac cells against irinotecan- and DOX-induced toxicity [32].

Our histopathological study confirmed our biochemical results by showing that DOX treatment disrupted normal cardiac architecture and caused fragmented cardiac muscle fibers in multiple areas. However, after the addition of DIL to DOX treatment, light microscopy showed normally branched cardiac muscle fibers with central vesicular nuclei and normal cardiomyocytes (Fig. 4a–d).

Overall, we have shown that DIL treatment increased cytotoxic effects of DOX against the growth of MCF-7 cells when given simultaneously, by induction of cell cycle arrest, promoting cell apoptosis, induction expression of tumor suppressor genes as FOXO3a and p53as well as reverse resistance gene expression. In the other side, the cardiotoxicity induced by DOX was attenuated in the presence of DIL.

## Conclusions

Our results suggest that addition of DIL may consider as a possible approach to enhance the cytotoxic activity of DOX against the growth of breast cancer cells by sensitizing its action, delay tumor growth through increased the activity of the tumor suppressor genes FOXO3a and p53 as well as ability to overcome the cell resistance through inhibition of P-gp expression. In addition, we also found that DIL treatment prevented DOX-induced cardiotoxicity in waster rat through its antioxidant properties that showed an increase in the level of GPx and TAC as well as a decreased in the level of MDA.

## Data Availability

The datasets used and/or analyzed during the current study are available from the corresponding author on reasonable request.
